# Stereotactic radiotherapy for ventricular tachycardia: A study protocol

**DOI:** 10.12688/f1000research.138758.2

**Published:** 2023-12-12

**Authors:** Mariko Kawamura, Masafumi Shimojo, Yasuya Inden, Takeshi Kamomae, Kuniyasu Okudaira, Tomohiro Komada, Sumire Aoki, Yurika Shindo, Ryotaro Yasui, Yusuke Yanagi, Masayuki Okumura, Takehiro Yamada, Yuka Kozai, Yumi Oie, Yutaka Kato, Shunichi Ishihara, Toyoaki Murohara, Shinji Naganawa

**Affiliations:** 1 Radiology, Nagoya University Hospital, Nagoya, Aichi Prefecture, Japan; 2 Cardiology, Nagoya University Hospital, Nagoya, Aichi Prefecture, Japan; 3 Radiological Technology, Nagoya University Hospital, Nagoya, Aichi Prefecture, Japan

**Keywords:** Radioablation, Stereotactic Radiotherapy, Stereotactic Radioablation, Ventricular Tachycardia (VT)

## Abstract

**Background:**

Currently, the standard curative treatment for ventricular tachycardia (VT) and ventricular fibrillation (VF) is radiofrequency catheter ablation. However, when the VT circuit is deep in the myocardium, the catheter may not be delivered, and a new, minimally invasive treatment using different energies is desired.

**Methods:**

This is a protocol paper for a feasibility study designed to provide stereotactic radiotherapy for refractory VT not cured by catheter ablation after at least one catheter ablation. The primary end point is to evaluate the short-term safety of this treatment and the secondary endpoint is to evaluate its efficacy as assessed by the reduction in VT episode. Cyberknife M6 radiosurgery system will be used for treatment, and the prescribed dose to the target will be 25Gy in one fraction. The study will be conducted on three patients.

**Conclusion:**

Since catheter ablation is the only treatment option for VT that is covered by insurance in Japan, there is currently no other treatment for VT/VF that cannot be cured by catheter ablation. We hope that this feasibility study will provide hope for patients who are currently under the stress of ICD activation.

**Trial registration:**

The study has been registered in the Japan Registry of Clinical Trials (jRCTs042230030).

## Introduction

Sustained ventricular tachycardia (VT) and ventricular fibrillation (VF) are arrhythmias that significantly reduce blood circulation and cause sudden cardiac death.

Treatment of patients with VT involves electrical cardioversion or implantable cardioverter defibrillator (ICD) implantation followed by administration of medication. Specially in patients with recurrent ventricular arrhythmias, it is essential to wear an ICD to continuously monitor cardiac activity and to restore a normal pulse with an electrical defibrillator when VT or VF is detected. Although ICD therapy is considered the most reliable treatment for preventing sudden death, frequent VT attacks and electroshock therapy with ICDs have been reported to worsen patients’ quality of life and long-term prognosis. ^1^^,^^2^

Currently, the standard curative treatment for VT and VF is radiofrequency catheter ablation with a three-dimensional (3D) mapping system. The conventional ablation therapy involves ablation from the endocardial side; however, in the case of ablation of the VT, ablation from the epicardial side may be performed depending on the thickness and location of the myocardial tissue to be ablated. However, a previous study has reported that more than half of the VT cases after myocardial infarction or other congenital cardiac diseases recur within one year of ablation. ^3^ Furthermore, although catheter ablation therapy clearly reduces the recurrence rate of VT, improvement in life expectancy remains unconfirmed. A key factor is the high recurrence rate, and improvements in treatment techniques are warranted.

The causes of tachyarrhythmia include arrhythmias due to abnormal automaticity and re-entry through the tachycardia circuit. To interrupt the tachycardia circuit, catheter ablation is performed as a curative treatment for arrhythmias caused by re-entry. Catheter ablation generates Joule heat in the myocardial tissue around the catheter electrode and cauterizes the tissue, thereby interrupting the tachycardia circuit. However, when the VT circuit is deep in the myocardium, under epicardial fatty tissues, or near the coronary arteries, the catheter may not be delivered, and a new, minimally invasive treatment using different energies is desired.

Since 2014, several case reports on stereotactic radiotherapy for VT have been published. ^4^^–^^9^ A systematic review published in 2021 revealed the safety and short-term reduction in sustained VT/VF burden. ^10^ To implement this method in Japan, the technique needs to be converted to one that can be implemented in Japan, and the safety of the method should be clarified in Japanese patients.

This study aimed to convert the results of overseas research into a feasible method in local practice and to verify whether radioablation using stereotactic radiotherapy techniques for patients with VT not cured by catheter ablation can reduce the number of VT occurrences compared to before radioablation treatment and whether the use of antiarrhythmic drugs can be reduced.

## Protocol

### Study design and patient eligibility

This prospective, single-arm, single-center study was approved by the Nagoya University ethics committee (CRB4180004) and registered to the Japan Registry of Clinical Trials (jRCTs042230030). Patients who are refractory to at least one catheter ablation and had received one or more anti-arrhythmia drug treatment with over three episodes of VT episode with therapeutic intervention (anti-tachycardia pacing (ATP) or shock) in the last three months are considered eligible to participate in the study. Because data from Asian countries are very limited, we will first design a feasibility study at a single center. The inclusion criteria are as follows: age of ≥18 years; received antiarrhythmic drug treatment after ICD implantation; with recurrent VT after at least one catheter ablation; have experienced ≥3 VT attacks within the past three months while taking at least one antiarrhythmic drug; with VT that cannot be treated with catheter ablation; without history of cardiac irradiation; and provided written informed consent.

Meanwhile, the exclusion criteria are as follows: with heart failure requiring a left ventricular assist device; with a low probability of survival of >6 months because of factors other than VT; with contraindications for irradiation; undergoing maintenance hemodialysis or peritoneal dialysis; with organic heart disease requiring surgery; with acute coronary or heart disease requiring hospitalization; at a high risk of radiation pneumonitis (e.g. idiopathic pulmonary fibrosis); with difficult-to-control pericardial or pleural effusions; having difficulty in resting in supine for 1 h; and with other illnesses or reasons that make it difficult to perform necessary examinations or follow-up investigations, or for which the principal investigator/participating physician determines that the patient is unfit to participate in this study. The discontinuation criteria are as follows: withdraws consent, or no longer meets the eligibility criteria while on the treatment waiting list, or difficult to continue the research due to an adverse event, or Accredited Clinical Research Review Committee instructs to discontinue the research, or the principal investigator/participating physician determines that the patient is unfit to participate in this study.

Any additional standard treatment of VT is acceptable. Changes in medication will be always monitored during the study. Medications will not be restricted if necessary. However, medications will be monitored throughout the study even if they are not considered directly relevant to the study.

### Endpoints

The primary endpoint is the short-term safety of the treatment. Safety evaluations will be performed on days 0, week 2, 1 month, 1.5-month, 2 month, 3 month, 4 month, 5 month, 6 month, 9 month, and 12 months after treatment. Myocardial dysfunction (e.g., contractility, diastolic dysfunction, pericarditis, pericardial effusion, myocardial ischemia, and heart failure), lung injury, and other treatment-related acute and subacute (up to 6 months after irradiation) complications greater than G3 in the Common Terminology Criteria for Adverse Events ver. 5.0 will be monitored. The secondary endpoint is the efficacy of the treatment. Cardiac function assessed by imaging, reduction of VT treatment (ATP and/or shock) by ICD, presence of dose reduction of antiarrhythmic drugs, overall survival, and patient quality of life will be measured using the 36-Item Short Form Survey and EuroQol-5D. Data collected for assessment of endpoints are as shown in Table 1. All data are registered to Research Electronic Data Capture (REDCap) at required timing as described previously. As this is the first treatment of VT using CyberKnife in Japan, we will evaluate the safety and efficacy of the treatment in three patients. If a patient enrolled in the study is not treated after enrollment, the patient is not counted, and individual enrollment will be continued until three eligible cases are treated. The enrollment will be terminated upon accumulation of three eligible cases.

**Table 1.** Timing and exams of follow up.

	Base line	Day0	Wk2	1M	1.5M	2M	3M	4M	5M	6M	9M	12M
ICD record (ATP and Shock)	○	○	○	○	○	○	○	○	○	○	○	○
Holter ECG (24hr)	○	○		○			○			○		○
Echocardiography	○			○			○			○		○
12-lead ECG	○		○	○	○	○	○	○	○	○	○	○
Blood test	○	○	○	○	○	○	○	○	○	○	○	○
Chest CT	○						○			○		○
Chest Xp	○	○		○		○		○	○		○	
Cardiac MRI	○			○								○
Myocardial scintigraphy	○			○								○
QOL check	○			○								○
Adverse event		○	○	○	○	○	○	○	○	○	○	○

ECG: electrocardiogram, ATP: anti-tachycardia pacing.

### Study plan

*Target delineation and planning*

Patients will undergo expiratory and inspiratory breath-holding and respiratory-gated contrast-enhanced computed tomography (CT) to create a stereotactic body radiotherapy (SBRT) plan. A 3D map from the electroanatomic mapping system (CARTO; Biosense-Webster, Israel) and ECG-gated CT scan will be fused to the planning CT to locate the target region and calculate the motion according to the heartbeats. The precision treatment system and CyberKnife M6 radiosurgery system (both from Accuray Inc., USA), which contain Synchrony, a respiratory compensation technology, are used to plan and deliver the planned dose. ^11^ Positron emission tomography/CT and contrast-enhanced magnetic resonance imaging could be used as secondary reference images. ICD leads are used for respiratory tracking. A 3-mm planning target volume (PTV) margin will be added to the target, and a dose of 25 Gy will be delivered to the PTV in a single session. The dose constraints are listed in Table 2. The PTV margin can be varied to account for setup errors and motion uncertainty, and the PTV dose can be reduced to 20 Gy to prioritize dose constraints for organs at risk. The maximum allowable dose is 32.5 Gy.

**Table 2.** Dose constraints.

Organ at risk	Max dose	Volume recommendation
trachea	20.2 Gy	V10.5 Gy < 4 cc
esophagus	15.4 Gy	V9 Gy < 5 cc
stomach	12.4 Gy	V11.2 Gy < 10 cc
aorta	25 Gy	<20 Gy preferred if possible
lung	-	V7.4 Gy < 1000 cc V7 Gy < 1500 cc
heart (exclude PTV)	25 Gy	V16 Gy < 15 cc Or D50% < 5 Gy
rib	30 Gy	V22 Gy < 1 cc
colonary artery	25 Gy	<14 Gy preferred if possible
atrium	4.4 Gy	preferred if possible
valve	10 Gy	Secondary: <25 Gy
spinal canal	8 Gy	
skin	14.4 Gy	V10 Gy < 10 cc
ICD	0.5 Gy	Does not allow the beam to pass through the ICD body

*Treatment*

The treatment as an inpatient procedure for at least 24 h after irradiation will be performed by a cardiologist and a radiation oncologist. During treatment, the ICD will be turned off, while the ECG will be carefully monitored by a cardiologist. The ICD will be turned on after completion of irradiation following an equipment check.

After treatment, a planned follow-up will be performed as described in the endpoint section.

*Quality control and quality assurance*

Monitoring will be conducted in accordance with the monitoring protocol and plan separately prescribed by the monitoring supervisor. (On-site monitoring will be conducted at least three times for each patient.) Documentation monitoring will be conducted twice during on-site monitoring, once at the beginning of the study and once at the end of the study. The audit will be conducted in accordance with the audit protocol and audit plan separately specified by the person in charge of the audit. (One system audit and one case audit will be conducted on-site.)

### Dissemination

The study is registered and is open in the Japan Registry of Clinical Trials (jRCTs042230030). The outcome of the study will be published in jRCTs.

### Study status

Participant recruitment started

## Discussion

Catheter ablation is the only curative treatment for VT and is covered by insurance in Japan. Catheter ablation allows treatment while directly searching for the origin using intracardiac electrocardiography and is undoubtedly a logical treatment method. However, because it uses Joule heating from the tip of the catheter, arrhythmias originating deep in the myocardium cannot be treated. Thus, VT requiring treatment of the deep myocardium, which is a weakness of catheter ablation, requires a different energy source than catheters, and there are great expectations for radioablation using SBRT.

Since experimental animals have shown that reducing the dose to 32 Gy or less can produce an antiarrhythmic effect in the short term without causing fibrosis, ^12^ this protocol was designed with an upper limit on the irradiation dose. At this point, however, there is some debate as to whether it is more effective to promote fibrosis with a higher target dose to obtain antiarrhythmic effects in the long term, and we hope that setting a maximum dose in this clinical trial will provide an answer to this part of the question.

To provide completely noninvasive treatment, treatment plans have been developed and implemented using epicardial electrocardiography at leading institutions; however, using this device in Japan is difficult because it is not approved in the country. Because catheter ablation has a smaller ablation area than radioablation, and although this narrower ablation area may be the cause of VT recurrence, there are many uncertainties, such as the long-term effect of irradiating a wider area on cardiac function.

In deference to catheter ablation, which has more long-term data than radioablation, we will change the energy to be irradiated from Joule heat to radiation dose based on the findings obtained with catheter ablation. It is highly possible that the mechanism of antiarrhythmic effect of irradiation is different from that of ablation, and furthermore, the mechanism may be different between ischemic and non-ischemic diseases. Therefore, it is essential to continue radioablation trial with prospective manners.

The facility where this study will be conducted has experience in treating >50 patients with VT with catheter ablation annually, which is a very high volume in Japan. Therefore, although this will be a single-center study, we believe that the facility is appropriate for evaluating resistance to catheter ablation. As no other treatment options are available for VT and VF, this study may serve as a guide to help clinicians in treating patients who failed to catheter ablation. Furthermore, we hope that this feasibility study will provide hope for patients who are currently under the stress of ICD activation.

### Ethical considerations

Ethics approval and consent to participate: The study was approved by the appropriate institutional ethics committee (CRB4180004) and registered to the Japan Registry of Clinical Trials ( jRCTs042230030) (registration date 06/02/2023, protocol ver. 1.2).

## Data Availability

No data are associated with this article.
