# Supplementary material for: Stereotactic radiotherapy for ventricular tachycardia: A study protocol
Source: F1000Res. 2023 Dec 12;12:798. Originally published 2023 Jul 10. [Version 2] doi: 10.12688/f1000research.138758.2 (PMC10776963; doi:10.12688/f1000research.138758.2)
Supplement: Peer review history

# Reviewer report: Reviewer response for version 2

**DOI:** 10.5256/f1000research.159914.r229540

**Published:** 2024-01-09

**Authors:** Ji Hyun Chang

**Affiliations:** 1 Department of Radiation Oncology, Seoul National University College of Medicine, Seoul, South Korea

The authors provided a clear and informative response to the comments. I hope the clinical trial progresses successfully.

Is the study design appropriate for the research question?

Yes

Is the rationale for, and objectives of, the study clearly described?

Yes

Are sufficient details of the methods provided to allow replication by others?

Yes

Are the datasets clearly presented in a useable and accessible format?

Yes

Reviewer Expertise:

radiotherapy

I confirm that I have read this submission and believe that I have an appropriate level of expertise to confirm that it is of an acceptable scientific standard.

# Reviewer report: Reviewer response for version 2

**DOI:** 10.5256/f1000research.159914.r229541

**Published:** 2023-12-19

**Authors:** Lukas Knybel

**Affiliations:** 1 Oncology, University Hospital Ostrava, Ostrava, Czech Republic; 2 Faculty of Medicine, University of Ostrava, Ostrava, Czech Republic

Thanks to the authors for the implementation of the required minor changes.

Is the study design appropriate for the research question?

Yes

Is the rationale for, and objectives of, the study clearly described?

Partly

Are sufficient details of the methods provided to allow replication by others?

Partly

Are the datasets clearly presented in a useable and accessible format?

Not applicable

Reviewer Expertise:

Radiation oncology- SBRT and SRS. Experience with SBRT of VT.

I confirm that I have read this submission and believe that I have an appropriate level of expertise to confirm that it is of an acceptable scientific standard.

# Reviewer report: Reviewer response for version 1

**DOI:** 10.5256/f1000research.151984.r197297

**Published:** 2023-11-16

**Authors:** Lukas Knybel

**Affiliations:** 1 Oncology, University Hospital Ostrava, Ostrava, Czech Republic; 2 Faculty of Medicine, University of Ostrava, Ostrava, Czech Republic

Thank you for the opportunity to review this paper. SBRT of the heart is still a relatively new method. Valid data from prospective studies are limited and therefore this project is considered to be resonable.

 The introductory part is comprehensive, however, I have a few minor comments. Some sentences from the 1st paragraph seem redundant in relation to cardiac SBRT.

 The 2nd paragraph contains a minor inaccuracy - there are also primo ICD implantations. 

 5th paragraph - To the best of our knowledge, the first reports in vivo studies were published in 2014-2015 ^1^^,^^2^.

 Study design and patient eligibility - VT attacks should be described more clearly. Anti arrhythmic drugs are meant with or without dose escalation?

 Endpoints section - How will the safety evaluation be performed? Clinically or imaging methods, etc.? Is the follow-up interval too frequent? How will monitoring be carried out (Clinically, laboratory, imaging methods)?

 Study plan section - PET  and MR imaging could be used as reference images - We think they will be used as a secondary series, the reference series will be CT.

 Dose constraints - related to the report of TG101 you listed the dose limits for smaller airways, not large bronchus.

Is the study design appropriate for the research question?

Yes

Is the rationale for, and objectives of, the study clearly described?

Partly

Are sufficient details of the methods provided to allow replication by others?

Partly

Are the datasets clearly presented in a useable and accessible format?

Not applicable

Reviewer Expertise:

Radiation oncology- SBRT and SRS. Experience with SBRT of VT.

I confirm that I have read this submission and believe that I have an appropriate level of expertise to confirm that it is of an acceptable scientific standard, however I have significant reservations, as outlined above.

## References

[1] : Stereotactic ablative radiotherapy for the treatment of refractory cardiac ventricular arrhythmia. *Circ Arrhythm Electrophysiol* .2015;8(3) : 10.1161/CIRCEP.115.002765 748-50 10.1161/CIRCEP.115.002765 26082532

[2] : Cardiac Radiosurgery for Malignant Ventricular Tachycardia. *Cureus* .2014; 10.7759/cureus.190 10.7759/cureus.190

# Reviewer report: Reviewer response for version 1

**DOI:** 10.5256/f1000research.151984.r216046

**Published:** 2023-10-24

**Authors:** Ji Hyun Chang

**Affiliations:** 1 Department of Radiation Oncology, Seoul National University College of Medicine, Seoul, South Korea

I am pleased to review Japan's inaugural clinical trial protocol. I eagerly anticipate a successful progression of this clinical research, with hopes of making a valuable contribution to the field.

 Minor points: 

 Regarding the exclusion criteria within the protocol, it appears that '<6 months' would be more appropriate to denote a low probability of survival due to factors other than VT, rather than '>6 months.'

 Specifying a maximum allowable dose may not be deemed necessary. Some of the researchers intentionally increase the dose within the GTV. 

 In the second paragraph of the discussion, the transition from epicardial ablation to SBRT appears somewhat incongruous.

Is the study design appropriate for the research question?

Yes

Is the rationale for, and objectives of, the study clearly described?

Yes

Are sufficient details of the methods provided to allow replication by others?

Yes

Are the datasets clearly presented in a useable and accessible format?

Yes

Reviewer Expertise:

radiotherapy

I confirm that I have read this submission and believe that I have an appropriate level of expertise to confirm that it is of an acceptable scientific standard, however I have significant reservations, as outlined above.

# Response

**Published:** 2023-11-09

**Authors:** Mariko Kawamura

**Affiliations:** Radiology, Nagoya University, Nagoya, Aichi Prefecture, Japan

Thanks for your very kind and thoughtful comments. I found them very helpful. I will briefly respond to you below.

 I agree that minimally invasive therapy is preferable for patients who are unlikely to survive long term for reasons other than VT. However, since this is a feasibility study, we considered it difficult to fully evaluate the safety of this treatment in patients for whom long-term survival is unlikely for reasons other than VT, and therefore we included patients who are likely to survive for more than 6 months.

 As for the upper limit of the dose, it may depend on the target size, but I am sure that your point is true, and I understand the idea that there is no need to set an upper limit. Based on the results of this study, we hope to have an answer in the future as to whether an upper limit is really necessary or rather the target dose should be higher.

 Since the history of SBRT is a little shallower than that of catheter ablation, we have kept a low profile. As the long-term results of SBRT become clearer, we hope to be able to recommend SBRT a little more strongly.
